# MicroRNA-15b regulates reversion-inducing cysteine-rich protein with Kazal motifs (RECK) expression in human uterine leiomyoma

**DOI:** 10.1186/s12958-016-0180-y

**Published:** 2016-08-17

**Authors:** Yichun Guan, Lankai Guo, Lawrence Zukerberg, Bo R. Rueda, Aaron K. Styer

**Affiliations:** 1 Center for Reproductive Medicine, The Third Affiliated Hospital of Zhengzhou University, Zhengzhou, 450052 Henan Province China; 2 Vincent Department of Obstetrics and Gynecology, Vincent Center for Reproductive Biology, Massachusetts General Hospital, Their 9, 55 Fruit Street, Boston, MA 02114 USA; 3 Department of Pathology, Massachusetts General Hospital, Boston, MA 02114 USA; 4 Departments of Obstetrics, Gynecology, and Reproductive Biology, Harvard Medical School, Boston, MA 02115 USA

**Keywords:** Uterine leiomyoma, Fibroids, miR-15b, RECK, Myometrium

## Abstract

**Background:**

Human uterine leiomyoma (fibroids; LYO) are the most common benign neoplasms in reproductive-aged women. Dysregulated extracellular matrix and irregular LYO reversion-inducing cysteine-rich protein with Kazal motifs (RECK) expression are thought to be mediated by aberrant microRNA (miR) expression. The relationship of miR-15b and RECK expression in LYO has not been studied.

**Methods:**

The expression levels of miR-15b and RECK were determined by quantitative RT-PCR, Western blot, and immunohistochemistry in cultures derived from commercial primary leiomyoma (cpLYO) and myometrial (cpMYO) cell lines and leiomyoma (pLYO) and myometrium (pMYO) tissue from surgical samples respectively. The relationship between miR-15b and RECK expression in cpLYO and pLYO (compared to their respective myometrial controls) was evaluated following transfection of cell cultures with either miR-15b mimic or inhibitor.

**Results:**

Elevated levels of miR-15b were observed in cpLYO (2.82-fold; *p* = 0.04) and pLYO cell (1.30-fold; *p* = 0.0001) cultures respectively compared to corresponding MYO cell controls. Following transfection with miR-15b mimic, cpLYO cells (0.62-fold; *p* < 0.0001) and pLYO cells (0.68-fold; *p* < 0.0001) demonstrated reduced RECK protein expression. Following transfection with miR-15b inhibitor, cpLYO cells (1.20-fold; *p* < 0.0001) and pLYO cells (1.31-fold; *p* = 0.0007) demonstrated elevated RECK protein expression. RECK protein expression was reduced in pLYO tissues (0.73-fold; *p* < 0.0001) and pLYO (0.47-fold; *p* = 0.047) cells when compared to the corresponding MYO tissue controls.

**Conclusion:**

Our findings suggest that miR-15b negatively regulates RECK expression in LYO, and increased miR-15b and decreased RECK expression may contribute to the pathobiology of LYO. The functional significance of miR-15b and RECK expression warrants further investigation as potential therapeutic targets for the treatment of human LYO.

## Background

Human uterine leiomyoma (fibroids; LYO) are benign uterine smooth muscle tumors with an estimated overall incidence of 75 % among reproductive-aged women, and are symptomatic in approximately 25 % of women [1–3]. This gynecologic disorder may result in heavy and painful menstrual bleeding, pelvic pressure and pain, recurrent pregnancy loss, and infertility. Hysterectomy is the only curative treatment for this condition, and the efficacy of medical therapy is inconsistent [4]. Fibroids are the leading indication for hysterectomy, and has been estimated to account for an annual healthcare expenditure of approximately 34 billion dollars [5]. It has been demonstrated that the ovarian steroid hormones, estrogen (E) and progesterone (P) may play a functional role in the growth and progression of LYO [6]. However, the exact mechanisms that contribute to fibroid development remain unclear. Genetic alterations, epigenetic mechanisms (DNA methylation and histone modifications), growth factors, cytokines, chemokines variations, and extracellular matrix (ECM) components also have been identified to be involved in the initiation and development of LYO [4, 7, 8]. Recently, differential microRNA (miRNA) expression has been implicated as a possible contributor to the pathobiology of LYO [9].

MicroRNAs are a class of small noncoding, single-stranded RNAs, ranging from 19 to 24 nucleotides, and serve as regulators of targeted gene expression at the post-transcriptional level [10]. Specifically, miRNAs bind to the 3’-untranslated regions (3’-UTRs) of target mRNAs and suppress translation from mRNA to relative protein and/or reduce the stability of mRNA [11, 12]. These noncoding RNAs have been implicated as mediators in several physiologic and pathologic processes including inflammation, cell proliferation, differentiation, apoptosis, invasion, migration, drug resistance and angiogenesis [13, 14]. A particular miRNA of interest, miR-15b, has been implicated as a mediator of apoptosis by targeting the antiapoptotic gene *bcl*_*2*_ in gastric cancer cells, mesenchymal stem cells, and rat hepatic cells [14–16]. Overexpression of miR-15b has been observed as a modulator of angiogenesis, cellular migration, and invasion [13, 17]. Data from global qPCR array analyses (unpublished, currently under peer-review) of matched fibroid/myometrium primary tissue from our tissue repository showed that several miRNAs species were differentially expressed (upregulated or downregulated) in fibroids compared to myometrium. MiR-15b was noted to be upregulated in fibroids compared to myometrium (*p* < 0.0001). Investigators have also demonstrated that miR-15b induces apoptosis by targeting and suppressing Rab1A in human hepatocellular carcinoma both in vitro and in vivo [11]. Although, the functional role of miR-15b in tumorigenesis in cancer biology has been investigated, the role of this miRNA, and its regulation of gene targets, has not been examined in uterine fibroids.

Reversion-inducing cysteine-rich protein with Kazal motifs (RECK) was initially discovered because of its capacity of inducing reversion in ras-activated fibroblasts [18]. RECK expression can be ubiquitously detected in normal adult human tissues, which is required for normal embryogenesis, vasculogenesis and tissue remodeling [19, 20]. Accumulating evidence has revealed that RECK has the ability to regulate the angiogenic, extracellular matrix (ECM) formation, and invasive and metastatic activities of malignant cells both in vitro and in vivo [21]. Reduced expression or loss of RECK expression has been significantly correlated with poor prognosis in colorectal, breast, lung, pancreatic and hepatocellular carcinomas [20–24]. Several recent studies suggest that miR-15b, miR-16, miR-21, miR-221, miR-222, miR-372, miR-373, miR-92a and miR-182 can regulate the expression level of RECK by targeting its conserved sequences of 3’-UTRs in different diseases [25–27]. The regulation of RECK expression in uterine fibroids has not been studied previously and warrants further investigation.

Since miR-15b was upregulated in fibroids in our array analyses, RECK has been reported to regulate cellular processes and/or tumorigenesis in cell types other than uterine fibroids, and miR-15b regulation of RECK in fibroids has not been studied, further investigation of this miR may provide further insight into the pathobiology of fibroids. The objective of this study was to delineate the effect of miR-15b on RECK expression in uterine fibroids and myometrium.

## Methods

### Patients and tissue collection

Patients undergoing myomectomy or hysterectomy for uterine fibroids from 2010 through 2013 were recruited for banking trial and written consent was obtained per IRB approved protocol (# 2007P000341). A portion of the intraoperative surgical specimen (uterine fibroid, matched myometrium, and serum) was collected within 1 h, snap frozen in liquid N_2_ and stored at −80 °C. Fibroids with a size range of 2 to 6 cm in their greatest dimension were banked. Matched myometrial sample was obtained no less than 1 cm from fibroid capsule and processed in a similar fashion. Following tissue collection and banking, annotated clinical data was collected and de-identified. All patients were premenopausal women in the follicular phase of the menstrual cycle based on the date of last menstrual period and had not received any hormonal medications 6 months before their hysterectomy. Some of the tissue was processed for RNA and protein extraction, and some was fixed with 4 % paraformaldehyde for immunohistochemistry (IHC) study. Another portion was used for the isolation of pLYO cells and pMYO cells (both referred to as primary cells in this manuscript).

### Cell lines

Human uterine smooth muscle cells (also known as HUt-SMC [PromoCell, Heidelberg, Germany]),will be identified as commercial primary myometrial cells, and abbreviated as cpMYO) were derived from human myometrium and were grown in smooth muscle cell growth medium 2 and supplemented with 5 % fetal calf serum (PromoCell), 0.5 % epidermal growth factor (PromoCell), basic fibroblast growth factor (PromoCell), and insulin (PromoCell). Human uterine leiomyoma cells (GM10964, will be identified as commercial primary leiomyoma cells and abbreviated as cpLYO) were purchased from Coriell Institute for Medical Research (Camden, NJ, USA), and were cultured in Medium 199 (1x) (Gibco, Grand Island, NY, USA) and 15 % fetal bovine serum (FBS) (Gibco), heparin (Sigma-Aldrich, St. Louis, MO) and endothelial cell growth supplement (ECGS) (PromoCell). All cell cultures were incubated in 5 % CO_2_at 37 °C. Upon reaching 70–80 % confluence, the cells were passaged using 0.05 % trypsin-EDTA (Gibco).

### Primary cell cultures

Primary leiomyoma (pLYO) cells and primary myometrial (pMYO) cells were isolated from patient matched LYO and MYO tissues following the previously described protocols [28]. Cells were cultured in DMEM/F12 (1:1) (1x) (Gibco) containing 10 % FBS, 1 % Pen Strep (Gibco) and 0.7 % amphotericin B (Sigma-Aldrich). The cell cultures were maintained at 37 °C and 5 % CO_2_. Once the cells were approximately 70–80 % confluent, they were trypsinized and expanded.

### miRNA transfection

Mature mirVana™ miRNA mimic (miRNA-15b mimic), mirVana™ miRNA inhibitor (miRNA-15b inhibitor), mirVana™ miRNA mimic Negative Control #1 and mirVana™ miRNA inhibitor Negative Control #1 were purchased from Ambion (Ambion/ThermoFisher Scientific, Grand Island, NY) and used at a concentration of 10nM/well. Cells were transfected with either mirVana™ miRNA mimic negative control #1 or mirVana™ miRNA inhibitor negative control #1 at all data points. Cells were plated at a density of 1 × 10^5^ cells/well in 12 well plates. The transfection was conducted using Lipofectamine^TM^RNAi MAX (Invitrogen/Thermo Scientific) on the following day according to the manufacturer’s protocol. RNA/protein was extracted from the cells for quantitative RT-PCR (qRT-PCR) and Western blot assays at the indicated time points post transfection.

### RNA isolation and quantitative RT-PCR assay

Total RNA was extracted from cultured cells and human tissue with mirVana miRNA Isolation kit (Ambion/ThermoFisher Scientific) in accordance with the manufacturer's protocol. The quantity and quality of the isolated RNAs were assessed using the ND-1000 Spectrophotometer (NanoDrop Technologies, Wilmington, DE). To evaluate the expression level of miR-15b in tissues and cells, cDNA was prepared with Universal cDNA Synthesis Kit II (Exiqon, Woburn, MA, USA) according to the manufacturer’s instructions. qRT-PCR was performed in a total volume of 20 *μ*L, containing 100 ng cDNA, hsa-miR-15b LNA™ PCR primer (Exiqon) and ExiLENT SYBR® Green master mix (Exiqon) on Bio-Rad's QX100™ Droplet Digital™ PCR System (Bio-Rad Laboratories, Munich, Germany). MiR-30c was employed as the internal control. For RECK mRNA expression determination in tissues and cultured cells, cDNA was synthesized using SuperScript® VILO™ cDNA Synthesis Kit (Invitrogen/ThermoScientific). QRT-PCR was carried out on the Bio-Rad's QX100™ Droplet Digital™ PCR System using SsoAdvanced™ universal SYBR® Green supermix (Bio-Rad Laboratories, Munich, Germany) and the following primers (Invitrogen/ThermoScientific): RECK sense 5’-tccatctggagatccctgtc-3’ and anti-sense 5’-tgccagcaaaacaagaacag-3’. Actin was applied as the internal control. All reactions were incubated at 95 °C for 10 min as initial denaturation followed by 40 cycles of 95 °C for 15 s and 60 °C for 1 min. The relative expression level of miRNA-15 band RECK was analyzed with the comparative cycle threshold method (2^-ΔΔCT^). Preparations lacking RNA were used in place of the cDNA and applied as a negative control. All reactions were performed in triplicate.

### Western blot analysis

Protein was extracted with 1X RIPA lysis buffer (Upstate Biotechnology, Charlottesville, VA, USA), 1 mM PMSF (Sigma, St. Louis, MO, USA) and a protease inhibitor cocktail (Sigma) from cultured cells and tissues. The protein content was evaluated using the DC Protein Assay (Bio-Rad Laboratories, Munich, Germany) and using bovine serum albumin (BSA) as the standard. Equal amounts of total protein were boiled and separated on NuPAGE® 4–12 % Bis-Tris Gel (Life Technologies, Carlsbad, CA, USA) and then transferred onto a PVDF membrane (Bio-Rad). The membranes were blocked by incubating in Tris-buffered saline and Tween-20 (TBST) containing 5 % skim dry milk for 2 h before incubation with rabbit monoclonal antibody against human RECK (Cell Signaling Technology, Beverly, MA, USA) at the dilution of 1:1000 and mouse anti-human *β*-actin monoclonal antibody (Cell Signaling Technology) at the dilution of 1:1000 at 4 °C overnight. Following washing, the membranes were subsequently probed with respective secondary antibodies (horseradish peroxidase-conjugated goat anti-rabbit or anti-mouse IgG (Santa Cruz Biotechnology, Inc, Santa Cruz, CA, USA) diluted 1:10000 for 1 h at room temperature. The protein band signals were detected by using an Amersham™ ECL™ PrimeWestern blotting detection reagent (GE Healthcare, UK) after rinsing membranes with TBST 3 times according to the manufacturer’s instructions. The strips were finally scanned with ChemiDoc^TM^ XRS+ imaging System (Bio-Rad Laboratories). Quantification of bands intensities was conducted via Image J software (National Institute of Health, Bethesda, MD, USA).

### Immunohistochemical staining

Immunohistochemistry (IHC) was conducted in leiomyoma and matched myometrial tissues by following the manufacturer’s guidelines to assess the expression level of RECK. In brief, 5-*μ*m paraffin tissue section slides were baked in a drying oven, deparaffinized and hydrated through xylene and graded alcohol. Antigens were retrieved by boiling slides in unmasking solution (Dako Corporation, Carpinteria, CA, USA), and endogenous peroxidase activity was quenched by incubation in 3 % H_2_O_2_ for 10 min. To reduce nonspecific background staining, the slides were allowed to block with 5 % normal goat serum in PBS with 0.1 % Triton X for 1 h at room temperature. Subsequently, they were incubated with primary antibody (dilution: 1:50) in a humidified chamber at 4 °C overnight. The signal was detected using 3,3′-diaminobenzidine (DAB) chromogen (Dako Corporation). Prior to recording images, the slides were counterstained with Hematoxylin QS (Vector Laboratories, Burlingame, CA, USA), dehydrated with xylene, and mounted with VectaMount AQ (Vector Laboratories) for long-term preservation. Immunohistochemistry staining intensity was graded on a semi quantitative scale from 0 to 4 (0 indicates none; 1, weak; 2, intermediate; 3, strong; 4, extremely strong). Two independent investigators determined the staining intensity of positive RECK stained cells in 3 random fields per tissue section in a blinded manner.

### Statistical analysis

Statistical analysis was undertaken using GraphPad Prism 5 software (GraphPad Software, Inc., La Jolla, CA). Collected data were presented as the mean ± SD. Student’s two-tailed *t*-test, Mann–Whitney *U* test, or chi-square (*χ*^2^) test was applied appropriately to identify the statistical significance of observed differences between groups. In all tests, a value of *p* < 0.05 was considered significant.

## Results

### Increased expression of miR-15b in human LYO

**Fig. 1:**
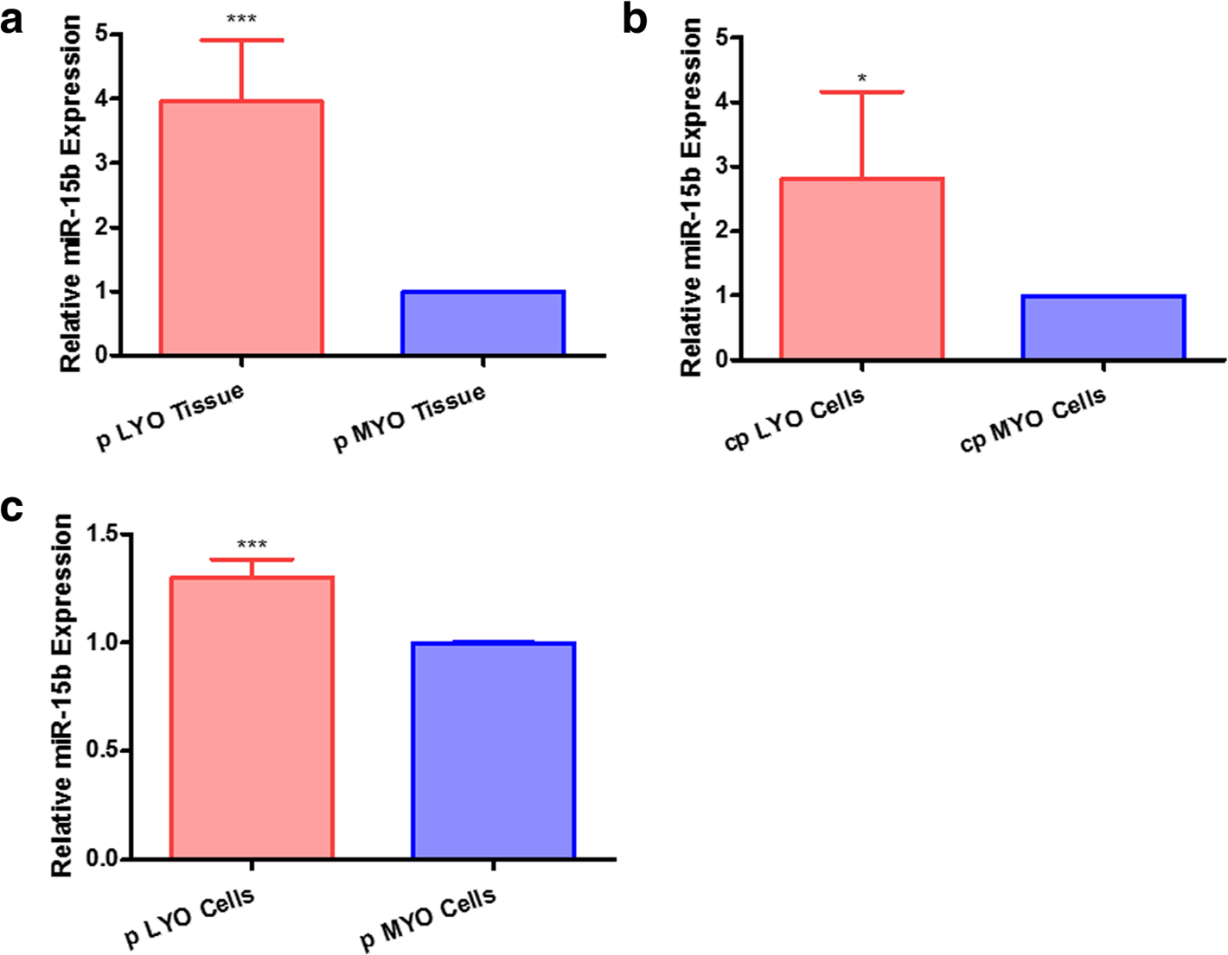
miR-15b expression is up-regulated in uterine leiomyoma tissue and cultured cells. **a** Comparison of miR-15b expression level between isolated pLYO tissue and paired pMYO tissue. **b** Expression level of miR-15b in cpLYO cells as well as cpMYO cells. **c** Comparison of miR-15b level between pLYO cells and pMYO cells. Quantitative RT-PCR assay was performed as described in the methods section. All data were presented as mean ± SD and analyzed using Student’s *t*-test (two tail, unequal variance) and Mann–Whitney *U* test applicable. The experiments were performed in triplicate. Statistical significance was noted as * *p* < 0.05, ** *p* < 0.005, *** *p* < 0.001

MiR-15b was observed to be differentially expressed between human leiomyoma and matched myometrial tissues in our miRNA microarray analysis (unpublished data). To confirm the preliminary results of miRNA microarray analysis, we compared miR-15b expression level in cells and tissues from leiomyoma and myometrial cells/tissues by qRT-PCR. Similar to the large scale miRNA microarray data, miR-15b expression was greater (3.95-fold; *p* < 0.0001) in pLYO tissues compared to paired pMYO tissues (Fig. 1a). Moreover, miR-15b was increased in cpLYO cell line (2.82-fold; *p* = 0.04, Fig. 1b) and pLYO cells (1.3-fold; *p* < 0.0001, Fig. 1c) when compared with their MYO cell controls, respectively.

### RECK is a potential target of miR-15b

**Fig. 2:**
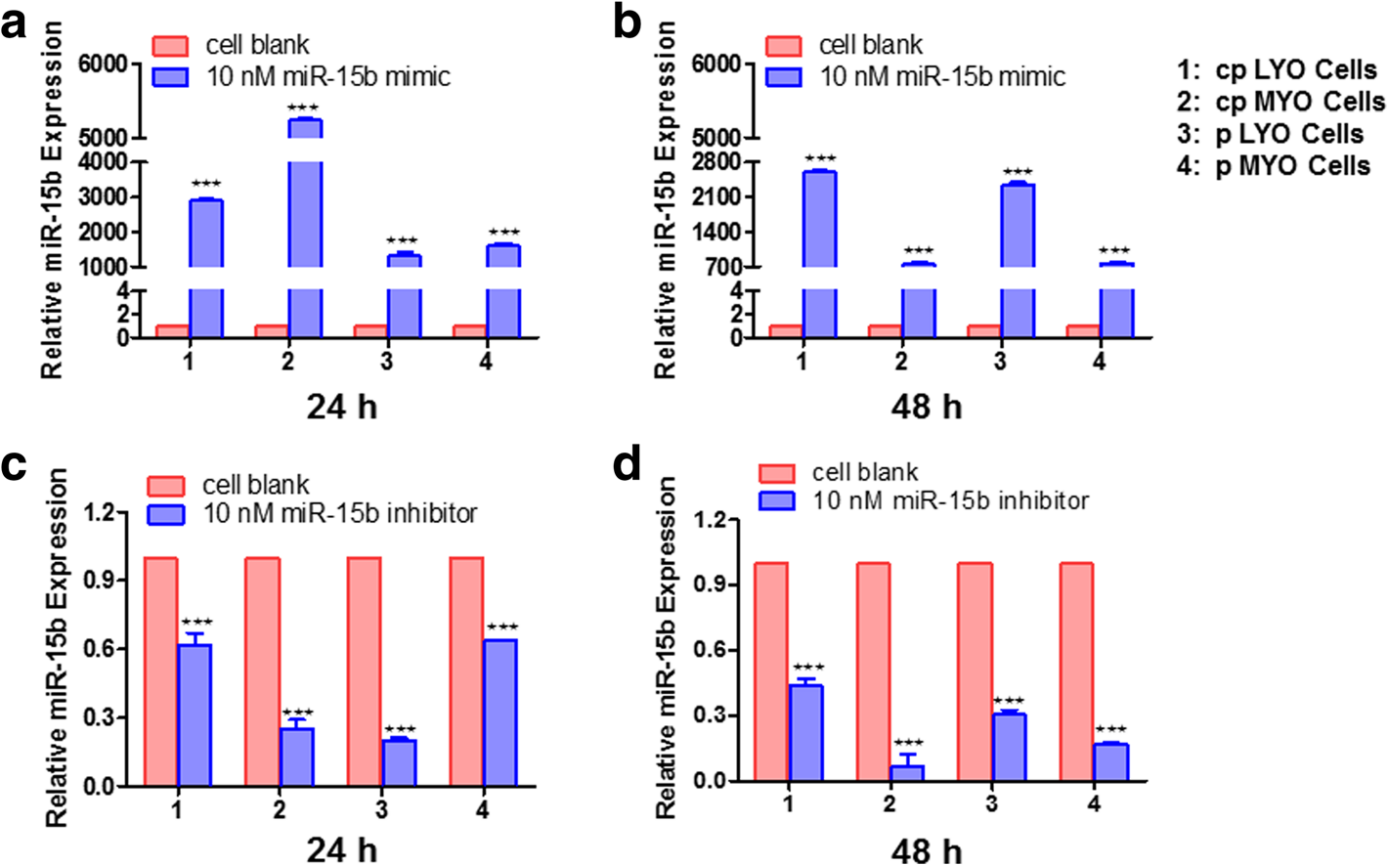
miR-15b mimic and inhibitor delivery into LYO and MYO cells via transient transfection. **a** Relative miR-15b expression in miR-15b mimic transfected cells 24 h post transfection. **b** Relative expression level of miR-15b in cells 48 h after miR-15b mimic transfection. **c** Influence of miR-15b inhibitor on miR-15b expression in cells 24 h after transfection. **d** Relative miR-15b expression in miR-15b inhibitor transfected cells 48 h post transfection. Cells without miR-15b mimic or miR-15b inhibitor transfection were used as control group, which was abbreviated as cell blank. Quantitative RT-PCR experiment was performed as described in methods. The data were presented as mean ± SD and analyzed using Student’s *t*-test (two tail, unequal variance) and Mann–Whitney *U* test appropriately. The experiments were conducted in triplicate. Statistical significance was noted as *** *p* < 0.001

Following transfection of cpLYO cell line, cpMYO cell line, pLYO cells and pMYO cells with miR-15b mimic, miR-15b expression was increased (*p* < 0.0001) by 2903.23, 5248.42, 1320.35 and 1607.57-fold 24 h in cpLYO cell line, cpMYO cell line, pLYO cells and pMYO cells, respectively (Fig. 2a). Following transfection with miR-15b inhibitor, miR-15b expression was reduced (*p* < 0.0001) by 0.62, 0.25, 0.20 and 0.64-fold at 24 h in cpLYO and cpMYO cell lines, pLYO cells and pMYO cells, respectively (Fig. 2c). Similar results were observed in miR-15b mimic and inhibitor treated cells respectively 48 h after transfection (Fig. 2b and d).

**Fig. 3:**
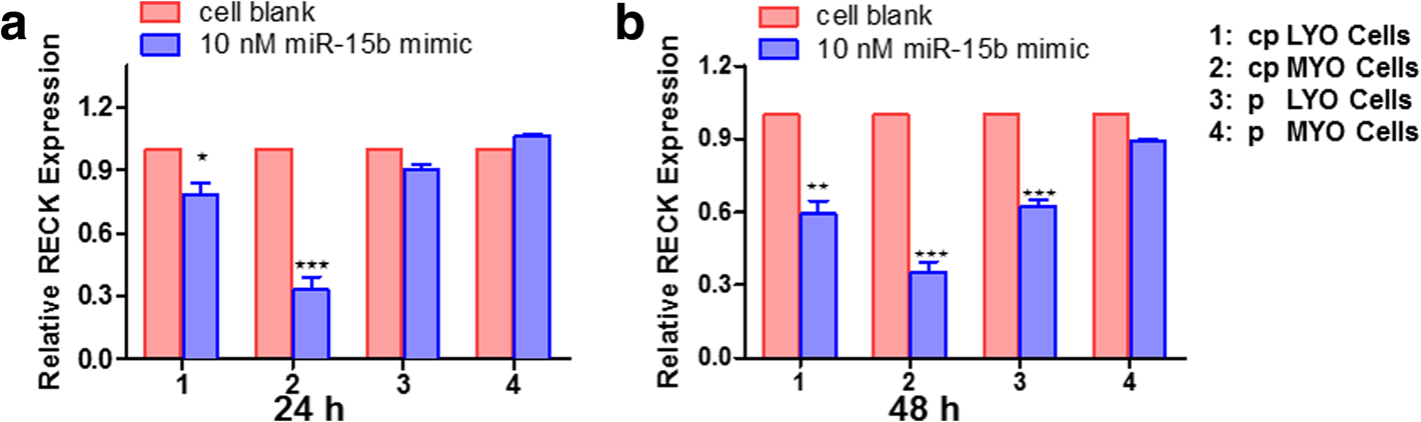
Inhibition of RECK mRNA expression by transfection miR-15b mimic in different type of cells. **a** qRT-PCR demonstrating decreased expression of RECK mRNA in cpLYO and cpMYO cells at 24 h post transfection with miR-15b mimic. **b** Reduction of RECK mRNA observed in miRNA-15b mimic transfected cpLYO, cpMYO and pLYO cells 48 h post transfection. Cells without miR-15b mimic transfection were used as control group, which was abbreviated as cell blank. Quantitative RT-PCR assay was performed as described in methods section. All data were presented as mean ± SD and analyzed using Student’s *t*-test (two tail, unequal variance) and Mann–Whitney *U* test when applicable. The experiments were performed in triplicate. Statistical significance was noted as* *p* < 0.05, ** *p* < 0.005, *** *p* < 0.001

To provide additional evidence that miR-15b may regulate RECK mRNA, the expression level of RECK mRNA was evaluated in miR-15b mimic transfected cells. RECK mRNA expression decreased in cpLYO cells (0.78-fold; *p* =0.03), cpMYO cells (0.33-fold; *p* < 0.0001), pLYO cells (0.90-fold; *p* = 0.27) and pMYO cells (1.07-fold, *p* = 0.96) respectively 24 h after miR-15b mimic transfection. Similarly, at 48 h post transfection, the RECK mRNA expression declined 0.59-fold (*p* = 0.003), 0.35-fold (*p* < 0.0001), 0.62-fold (*p* < 0.0001), and 0.89-fold (*p* = 0.22) in miR-15b mimic transfected cpLYO cells, cpMYO cells, pLYO cells and pMYO cells, respectively, when compared with cells without treatment (Fig. 3a and b).

### The expression level of RECK is decreased in primary leiomyoma tissues and cells

**Fig. 4:**
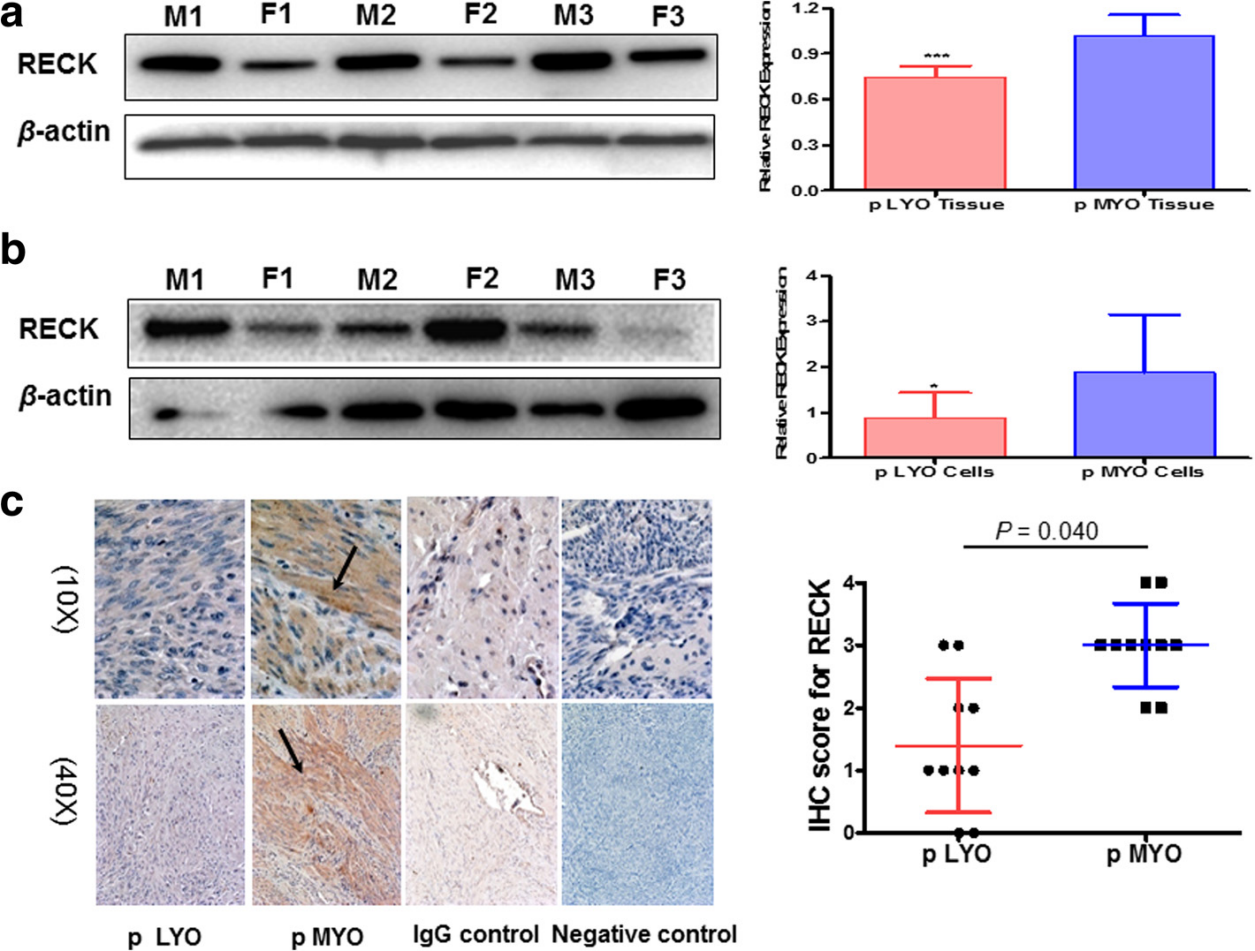
Differential expression level of RECK in pLYO and pMYO paired tissues and isolated cells. **a** RECK expression level in matched pLYO and pMYO tissues as determined by Western blot analysis. **b** Western blot analysis of RECK in pLYO and pMYO cell cultures from patient matched samples. **c** Representative matched pLYO and pMYO immunostained for RECK. The distribution of RECK immunohistochemical staining of paraffin sections from matched pLYO and pMYO tissue. Magnification: 10 × (*left panel*) and 40 × (*right panel*); *Arrows* indicated positive expression of RECK. Distribution of RECK immunohistochemical staining scores in matched LYO and MYO tissues were also showed. Data represented as Mean ± SD and analyzed using Student’s *t*-test (two tail, unequal variance), Mann–Whitney *U* test and chi-square (*χ* ^2^) test appropriately. Statistical significance was noted as * *p* < 0.05, ** *p* < 0.005, *** *p* < 0.001

We assessed the RECK protein expression in LYO and MYO tissues and primary cultured cells in the absence of mimics or inhibitors to determine base line comparisons of RECK protein in patient matched samples. Lower RECK protein expression was observed in pLYO tissues (0.73-fold; *p* < 0.0001) and pLYO cells (0.47-fold, *p* = 0.047) compared with that of pMYO tissues and pMYO cells, respectively (Fig. 4a and b). The relative RECK protein expression level was quantified by densitometry and the results were also shown in Fig. 4a and b. Immunohistochemistry was then carried out to further evaluate the expression level of RECK protein in tissue sections collected from pLYO and matched pMYO. Both cytoplasmic and nuclear immunoreactivity of RECK was observed, and specific RECK protein staining was primarily localized in cell cytoplasm. The immunostaining of RECK was visually less in the pLYO group compared with that in the matched pMYO group (Fig. 4c). The immunostaining intensities of RECK were assessed, and decreased RECK positive cells were observed in LYO tissues as compared with that in matched control specimens (0.47-fold; *p* = 0.04; Fig. 4c).

**Fig. 5:**
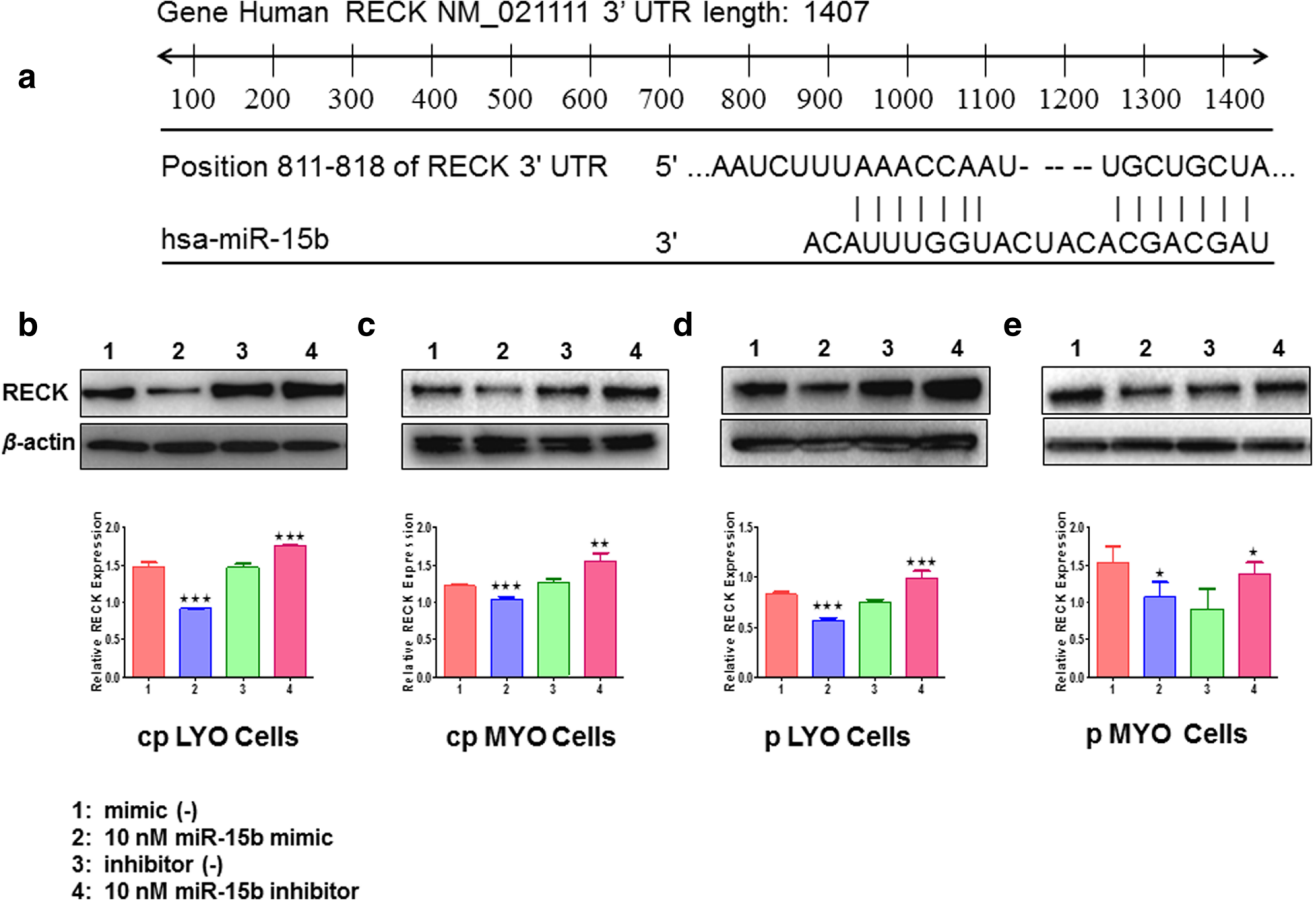
miR-15b is predicted to bind to the 3’-UTR of RECK and regulates RECK expression. **a** Scheme of the potential binding sites of miR-15b in the 3’-UTR of RECK. RECK mRNA has the potential complimentary binding site with miR-15b within its 3’-UTR. **b** miR-15b regulation of RECK protein expression in cpLYO cells. Proteins from cpLYO cells transfected with either miR-15b mimic or inhibitor at 10 nM. After 48 h, the lysates were analyzed by Western blot. **c** miR-15b regulation of RECK protein expression in cpMYO cells. **d** miR-15b regulation of RECK protein expression in pLYO cells. **e** miR-15b regulation of RECK protein expression in pMYO cells. Data were presented as means ± SD. Statistical comparisons between miR-15b mimic/inhibitor transfected cells and mimic/inhibitor negative control cells was analyzed using Student’s t-test (two tail, unequal variance) and Mann–Whitney *U* test appropriately. Statistical significance was noted as *p < 0.05, **p < 0.005, ***p < 0.001

Three publicly available bioinformatic algorithms, TargetScan, PicTar, and miRanda, http://www.targetscan.org; http://pictar.mdc-berlin.de/ and http://www.microrna.org/ were adopted to identify the potential target genes of miR-15b. Among these genes, RECK was selected as the candidate for further analysis since miR-15b can bind to the 3’-UTR of RECK. It has been predicted that miR-15b shared 8 identical nucleotides of the 5’ “seed” region that are complementary to bases 811–813 of the RECK 3’-UTR (Fig. 5a), and therefore it may potentially target RECK by in silico analysis [26]. To ascertain whether miR-15b modulate RECK expression, the cells were transfected with either specific miR-15b mimic or inhibitor at a final concentration of 10 nM for 48 h to increase or reduce endogenous miR-15b expression in both cell lines and cultured primary cells. A lower (0.62-fold; *p* < 0.0001) levels of RECK protein was observed in miR-15b mimic transfected cpLYO cells compared to mimic control transfected cells, while miR-15b knockdown by miR-15 inhibitor transfection resulted in increased (1.20-fold; *p* < 0.0001) RECK expression as determined by Western blot analysis (Fig. 5b). Lower (0.85-fold; *p* < 0.0001) levels of RECK protein was observed in miR-15b mimic transfected cpMYO cells compared to mimic control transfected cells, while miR-15b knockdown by miR-15 inhibitor transfection resulted in increased (1.22-fold; *p* = 0.003) (Fig. 5c). Similar results were exhibited in pLYO cells with miR-15b mimic transfection (0.68-fold; *p* < 0.0001) and inhibitor transfection (1.31-fold; *p* = 0.0007) (Fig. 5d). Similar results were found in pMYO cells by transfecting miR-15b mimic (0.70-fold; *p* = 0.02) and by transfecting miR-15b inhibitor (1.53-fold; *p* = 0.02) (Fig. 5e).

## Discussion

In the current study, we investigated the expression patterns of miR-15b and RECK in cultured primary LYO and MYO cells derived from commercial sources, and in primary LYO and MYO cells and tissue derived from hysterectomy samples from our patient population. Our findings demonstrate that miR-15b expression is elevated in leiomyoma compared to the myometrium. Conversely, RECK expression is lower in LYO tissues and cultured LYO cells (commercial and primary sources) when compared to myometrial controls. The inverse relationship in expression between miR-15b and RECK was also observed during transfection studies which overexpressed and inhibited miR-15b via mimics or antagomirs, respectively. To our knowledge, this is the first study to begin to delineate the possible functional significance of miR-15b as a regulator of RECK expression in fibroids.

MiR-15b is a member of the miR-15/16 superfamily, which possesses a 5’-end AGCAGC sequence, and includes miR-15a, miR-15b, miR-16, miR-195, miR-322, miR-497 among others. Overexpression of miR-15b has been correlated with poor prognosis and tumorigenesis. Several investigators have implied that miR-15b is a possible prognostic molecular marker and potential therapeutic target in cancer biology [23, 29]. Recent studies have reported overexpression of miR-15b exhibited in cells lines of human hepatocellular carcinoma, pancreatic cancer, acute promyelocytic leukemia, cervical cancer and malignant melanoma when compared to cell lines derived from benign tissue [11, 30, 31]. Marked upregulation in miR-15b expression was demonstrated to regulate the expression of targeted bcl-2, cyclin E1, Rab1A, NRP-2, MMP-3 and VEGFR-2, and therefore modulate numerous cellular biological processes, such as cell proliferation, division, apoptosis, migration, invasion, metabolism, stress, angiogenesis and drug resistance [11, 13–15, 23, 32, 33].

Previous studies have reported global miRNA expression patterns in paired sets of LYO and MYO tissue, and have described several differentially expressed miRNAs, including miR-15b. Unfortunately, the results of some studies are inconsistent with regards to the relative expression of miR-15b in LYO tissue compared to MYO tissue [9, 29, 34–37]. An analysis consisting of 213 human miRNAs probes identified that lower expression level of miR-15b was exhibited in leiomyomas as compared to their paired myometrium. However, expression levels of miR-15b were not different among matched isolated myometrial (MSMC) and leiomyoma (LSMC) smooth muscle cells, spontaneously transformed LSMC (t-LSMC) and a leiomyosarcoma cell line (SKLMS-1) [9, 35]. Applying comparative genomic hybridization analysis in 8 LYO of black women, it was demonstrated that miR-15b was lost in two of the 8 women [34]. In two other studies, differential expression of miR-15b was not shown in human LYO versus MYO in a microarray analysis containing of 454 miRNA species and one consisting of 206 human miRNAs [29, 36]. Moreover, Boryana Georgieva et al. used next-generation sequencing approaches and bioinformatics methods to determine the aberrant expressed miRNAs in LYO and MYO, and did not observe differential miR-15b expression [37]. In contrast to previous studies, we observed higher expression of miR-15b in LYO than their corresponding MYO controls following microarray and qRT-PCR. Possible explanations for variable results may be due to different techniques of miRNA analyses, which may include a wide range of array analyses with or without PCR validation of findings. From a demographic standpoint, differential genetics, and race/ethnicity of patients, fibroid sizes, inconsistent specimen processing techniques (from surgical sample) and intrinsic biologic variability may also contribute to variable results among studies.

Based on available bioinformatic algorithms that predict targets, RECK may be a target of miR-15b. Interestingly, a recent report also revealed that miR-15b holds the ability to negatively modulate targeted RECK in the HT1080 human fibrosarcoma cell line and Caco-2, a colon carcinoma cell line [26]. To our knowledge, the expression pattern of RECK had not been well established in human LYO relative to myometrium. This led us to explore this relationship. We demonstrated that RECK is expressed at lower level in LYO tissues than myometrial tissues as determined by Western blot and IHC. We also extend this observation to show that reduced relative levels of RECK expression was present in primary LYO cells as compared to primary MYO cells in this study. Our data further support this inverse relationship as evidenced in LYO and MYO cell cultures (both commercial and derived from primary tissue samples) following transfection of either miR-15b mimic or miR-15b inhibitor to either overexpress or inhibit miR-15b expression. The addition of miR-15 mimics resulted in decreased RECK. Likewise, transfection with miR-15b antagomirs increased RECK expression. These data provide evidence to suggest that miR-15b by indirect or direct interaction can regulate RECK expression.

RECK, a recently characterized membrane bound protein, has been identified to play a pivotal role in cancer progression by limiting invasion, metastasis and angiogenesis of tumors [19–21, 38, 39]. A large number of tumor cell lines and tissues including colorectal, breast, pancreatic, gastric cancer have low or undetectable RECK expression, [19, 25]. For example, decreased RECK expression has been reported in 81.8 % of 11 gastric cancer cell lines and 52 % of the 102 gastric cancer tissues [40]. Furthermore, accumulated evidence amply supports that RECK holds the potential value as a prognostic indicator for various human malignancies [22, 24, 38, 40–43]. Pancreatic cancer patients with higher RECK expression showed statistical better prognosis as compared to that with negative RECK expression [22]. Likewise, a study conducted in a cohort of 283 colorectal cancer patients implied that reduced RECK expression was an independent prognostic biomarker of poor survival [24]. There is also an obvious inverse correlation between RECK expression and macroscopic tumor growth, lymphatic invasion, lymph node metastasis as well as stage, which suggests that RECK constitutes a good prognostic marker in gastric cancer [40]. Therefore, if there were elevated levels of miR-15b in leiomyoma it may well down regulate the anti-tumor effects of RECK. Future studies which induce long-term over and under-expression of miR 15-b via lentiviral infection of a mimic and antagomir in fibroids and myometrium primary cell culture and immortalized cultures will be necessary to evaluate the possible anti-tumor effects of RECK by evaluating cell proliferation and the cell cycle transition via flow cytometry.

From another perspective, the most distinct characteristic of LYO is the excessive deposition of ECM and synthesis of ECM proteins, such as MMP-2, MMP-9, MMP-11 [44, 45]. MicroRNA-15 was previously reported to inhibit the expressions of MMP-2, MMP-9 and MMP-14 which involves in breaking down the ECM [46–49]. We therefore speculate that with increased miR-15b and reduced RECK expression, there may be reduced RECK mediated inhibition of ECM proteins synthesis, aberrant ECM production and an increased potential for LYO development. Whether miR-15b has the capacity to specifically regulate the breakdown of ECM or be involved in other cellular functional activities, such as apoptosis, angiogenesis, migration and invasion, in human LYO is yet to be determined. Future studies will be necessary to investigate if miR-15b overexpression in fibroid and myometrium studies result in upregulation of the fibroid relevant MMP, 2, 9, and 11 and increase production of type 1 and 3 fibrillar collagens, which are characteristic of the signature aberrant ECM production in this gynecologic disorder.

## Conclusion

Our findings demonstrate that overexpression of miR-15b results in concurrent decreased RECK expression in human LYO, and suggests that miR-15b may target RECK in LYO. Collectively, our results provide further insight into potential mechanisms for miR mediated regulation of fibroid development and/or progression and warrants further investigation.

## Abbreviations

LYO: Human uterine leiomyoma
miRNA: microRNA
cpLYO cells: Commercially prepared leiomyoma cells
cpMYO cells: Commercially prepared myometrial cells
pLYO cells: Freshly prepared primary leiomyoma cells
pMYO cells: Freshly prepared primary myometrial cells
3’-UTRs: 3’-untranslated regions
RECK: Reversion-inducing cysteine-rich protein with Kazalmotifs
E: Estrogen
P: Progesterone
ECM: Extracellular matrix
IHC: Immunohistochemistry
FBS: Fetal bovine serum
BSA: Bovine serum albumin
TBST: Tris-buffered saline and Tween-20
DAB: 3,3′-diaminobenzidine

## References

[1] Mehine M, Kaasinen E, Makinen N, Katainen R, Kampjarvi K, Pitkanen E (2013). Characterization of uterine leiomyomas by whole-genome sequencing. N Engl J Med.

[2] Fitzgerald JB, Chennathukuzhi V, Koohestani F, Nowak RA, Christenson LK (2012). Role of microRNA-21 and programmed cell death 4 in the pathogenesis of human uterine leiomyomas. Fertil Steril.

[3] Eltoukhi HM, Modi MN, Weston M, Armstrong AY, Stewart EA (2014). The health disparities of uterine fibroid tumors for African American women: a public health issue. Am J Obstet Gynecol.

[4] Commandeur AE, Styer AK, Teixeira JM (2015). Epidemiological and genetic clues for molecular mechanisms involved in uterine leiomyoma development and growth. Hum Reprod Update.

[5] Cardozo ER, Clark AD, Banks NK, Henne MB, Stegmann BJ, Segars JH (2012). The estimated annual cost of uterine leiomyomata in the United States. Am J Obstet Gynecol.

[6] Reis FM, Bloise E, Ortiga-Carvalho TM. Hormones and pathogenesis of uterine fibroids. Best Pract Res Clin Obstet Gynaecol. 2016;34:13–24. 10.1016/j.bpobgyn.2015.11.015 26725037

[7] Flake GP, Andersen J, Dixon D (2003). Etiology and pathogenesis of uterine leiomyomas: a review. Environ Health Perspect.

[8] Islam MS, Protic O, Stortoni P, Grechi G, Lamanna P, Petraglia F (2013). Complex networks of multiple factors in the pathogenesis of uterine leiomyoma. Fertil Steril.

[9] Karmon AE, Cardozo ER, Rueda BR, Styer AK (2014). MicroRNAs in the development and pathobiology of uterine leiomyomata: does evidence support future strategies for clinical intervention?. Hum Reprod Update.

[10] Ryan BM, Robles AI, Harris CC (2010). Genetic variation in microRNA networks: the implications for cancer research. Nat Rev Cancer.

[11] Yang Y, Hou N, Wang X, Wang L, Chang S, He K (2015). miR-15b-5p induces endoplasmic reticulum stress and apoptosis in human hepatocellular carcinoma, both in vitro and in vivo, by suppressing Rab1A. Oncotarget.

[12] Filipowicz W, Bhattacharyya SN, Sonenberg N (2008). Mechanisms of post-transcriptional regulation by microRNAs: are the answers in sight?. Nat Rev Genet.

[13] Zheng X, Chopp M, Lu Y, Buller B, Jiang F (2013). MiR-15b and miR-152 reduce glioma cell invasion and angiogenesis via NRP-2 and MMP-3. Cancer Lett.

[14] Xia L, Zhang D, Du R, Pan Y, Zhao L, Sun S (2008). miR-15b and miR-16 modulate multidrug resistance by targeting BCL2 in human gastric cancer cells. Int J Cancer.

[15] Guo CJ, Pan Q, Li DG, Sun H, Liu BW (2009). miR-15b and miR-16 are implicated in activation of the rat hepatic stellate cell: An essential role for apoptosis. J Hepatol.

[16] Yu B, Gong M, He Z, Wang YG, Millard RW, Ashraf M (2013). Enhanced mesenchymal stem cell survival induced by GATA-4 overexpression is partially mediated by regulation of the miR-15 family. Int J Biochem Cell Biol.

[17] Chan LS, Yue PY, Wong YY, Wong RN (2013). MicroRNA-15b contributes to ginsenoside-Rg1-induced angiogenesis through increased expression of VEGFR-2. Biochem Pharmacol.

[18] Takahashi C, Sheng Z, Horan TP, Kitayama H, Maki M, Hitomi K (1998). Regulation of matrix metalloproteinase-9 and inhibition of tumor invasion by the membrane-anchored glycoprotein RECK. Proc Natl Acad Sci U S A.

[19] Clark JC, Thomas DM, Choong PF, Dass CR (2007). RECK--a newly discovered inhibitor of metastasis with prognostic significance in multiple forms of cancer. Cancer Metastasis Rev.

[20] Meng N, Li Y, Zhang H, Sun XF (2008). RECK, a novel matrix metalloproteinase regulator. Histol Histopathol.

[21] Alexius-Lindgren M, Andersson E, Lindstedt I, Engstrom W (2014). The RECK gene and biological malignancy--its significance in angiogenesis and inhibition of matrix metalloproteinases. Anticancer Res.

[22] Masui T, Doi R, Koshiba T, Fujimoto K, Tsuji S, Nakajima S (2003). RECK expression in pancreatic cancer: its correlation with lower invasiveness and better prognosis. Clin Cancer Res.

[23] Oh J, Takahashi R, Kondo S, Mizoguchi A, Adachi E, Sasahara RM (2001). The membrane-anchored MMP inhibitor RECK is a key regulator of extracellular matrix integrity and angiogenesis. Cell.

[24] Stenzinger A, von Winterfeld M, Rabien A, Warth A, Kamphues C, Dietel M (2012). Reversion-inducing cysteine-rich protein with Kazal motif (RECK) expression: an independent prognostic marker of survival in colorectal cancer. Hum Pathol.

[25] Chen Y, Tseng SH (2012). The potential of RECK inducers as antitumor agents for glioma. Anticancer Res.

[26] Loayza-Puch F, Yoshida Y, Matsuzaki T, Takahashi C, Kitayama H, Noda M (2010). Hypoxia and RAS-signaling pathways converge on, and cooperatively downregulate, the RECK tumor-suppressor protein through microRNAs. Oncogene.

[27] Lin HY, Chiang CH, Hung WC (2013). STAT3 upregulates miR-92a to inhibit RECK expression and to promote invasiveness of lung cancer cells. Br J Cancer.

[28] Chang HL, Senaratne TN, Zhang L, Szotek PP, Stewart E, Dombkowski D (2010). Uterine leiomyomas exhibit fewer stem/progenitor cell characteristics when compared with corresponding normal myometrium. Reprod Sci.

[29] Wang T, Zhang X, Obijuru L, Laser J, Aris V, Lee P (2007). A micro-RNA signature associated with race, tumor size, and target gene activity in human uterine leiomyomas. Genes Chromosomes Cancer.

[30] Satzger I, Mattern A, Kuettler U, Weinspach D, Voelker B, Kapp A (2010). MicroRNA-15b represents an independent prognostic parameter and is correlated with tumor cell proliferation and apoptosis in malignant melanoma. Int J Cancer.

[31] Finnerty JR, Wang WX, Hebert SS, Wilfred BR, Mao G, Nelson PT (2010). The miR-15/107 group of microRNA genes: evolutionary biology, cellular functions, and roles in human diseases. J Mol Biol.

[32] Vimalraj S, Selvamurugan N (2015). Regulation of proliferation and apoptosis in human osteoblastic cells by microRNA-15b. Int J Biol Macromol.

[33] Wang X, Tang S, Le SY, Lu R, Rader JS, Meyers C (2008). Aberrant expression of oncogenic and tumor-suppressive microRNAs in cervical cancer is required for cancer cell growth. PLoS One.

[34] Zavadil J, Ye H, Liu Z, Wu J, Lee P, Hernando E (2010). Profiling and functional analyses of microRNAs and their target gene products in human uterine leiomyomas. PLoS One.

[35] Pan Q, Luo X, Chegini N (2008). Differential expression of microRNAs in myometrium and leiomyomas and regulation by ovarian steroids. J Cell Mol Med.

[36] Marsh EE, Lin Z, Yin P, Milad M, Chakravarti D, Bulun SE (2008). Differential expression of microRNA species in human uterine leiomyoma versus normal myometrium. Fertil Steril.

[37] Georgieva B, Milev I, Minkov I, Dimitrova I, Bradford AP, Baev V (2012). Characterization of the uterine leiomyoma microRNAome by deep sequencing. Genomics.

[38] Takeuchi T, Hisanaga M, Nagao M, Ikeda N, Fujii H, Koyama F (2004). The membrane-anchored matrix metalloproteinase (MMP) regulator RECK in combination with MMP-9 serves as an informative prognostic indicator for colorectal cancer. Clin Cancer Res.

[39] Sasahara RM, Brochado SM, Takahashi C, Oh J, Maria-Engler SS, Granjeiro JM (2002). Transcriptional control of the RECK metastasis/angiogenesis suppressor gene. Cancer Detect Prev.

[40] Song SY, Son HJ, Nam E, Rhee JC, Park C (2006). Expression of reversion-inducing-cysteine-rich protein with Kazal motifs (RECK) as a prognostic indicator in gastric cancer. Eur J Cancer.

[41] Takenaka K, Ishikawa S, Kawano Y, Yanagihara K, Miyahara R, Otake Y (2004). Expression of a novel matrix metalloproteinase regulator, RECK, and its clinical significance in resected non-small cell lung cancer. Eur J Cancer.

[42] Span PN, Sweep CG, Manders P, Beex LV, Leppert D, Lindberg RL (2003). Matrix metalloproteinase inhibitor reversion-inducing cysteine-rich protein with Kazal motifs: a prognostic marker for good clinical outcome in human breast carcinoma. Cancer.

[43] van der Jagt MF, Sweep FC, Waas ET, Hendriks T, Ruers TJ, Merry AH (2006). Correlation of reversion-inducing cysteine-rich protein with kazal motifs (RECK) and extracellular matrix metalloproteinase inducer (EMMPRIN), with MMP-2, MMP-9, and survival in colorectal cancer. Cancer Lett.

[44] Halder SK, Osteen KG, Al-Hendy A (2013). Vitamin D3 inhibits expression and activities of matrix metalloproteinase-2 and −9 in human uterine fibroid cells. Hum Reprod.

[45] Koohestani F, Braundmeier AG, Mahdian A, Seo J, Bi J, Nowak RA (2013). Extracellular matrix collagen alters cell proliferation and cell cycle progression of human uterine leiomyoma smooth muscle cells. PLoS One.

[46] Qin J, Luo M (2014). MicroRNA-221 promotes colorectal cancer cell invasion and metastasis by targeting RECK. FEBS Lett.

[47] Xin C, Buhe B, Hongting L, Chuanmin Y, Xiwei H, Hong Z (2013). MicroRNA-15a promotes neuroblastoma migration by targeting reversion-inducing cysteine-rich protein with Kazal motifs (RECK) and regulating matrix metalloproteinase-9 expression. FEBS J.

[48] Chiang CH, Hou MF, Hung WC (1830). Up-regulation of miR-182 by beta-catenin in breast cancer increases tumorigenicity and invasiveness by targeting the matrix metalloproteinase inhibitor RECK. Biochim Biophys Acta.

[49] Takagi S, Simizu S, Osada H (2009). RECK negatively regulates matrix metalloproteinase-9 transcription. Cancer Res.

